# A comprehensive inventory of communication tower infrastructure across the range of greater and Gunnison sage-grouse

**DOI:** 10.1038/s41597-026-07296-y

**Published:** 2026-05-02

**Authors:** Sarah C. Webster, Shawn Szabo, Jacqueline B. Cupples, Shawn T. O’Neil, Jonathan B. Dinkins, Steve Abele, Jennifer M. Hill, John C. Tull, Michael P. Chenaille, Peter S. Coates

**Affiliations:** 1 U.S. Geological Survey, Western Ecological Research Center, Reno Field Station, 1100 Valley Rd., Reno, NV 89512 USA; 2 U.S. Fish and Wildlife Service, Ecological Services, 1340 Financial Boulevard, Suite 234, Reno, Nevada USA; 3 Oregon State University, Department of Animal and Rangeland Sciences, 206 Withycombe, Corvallis, OR 97331 USA; 4 U.S. Fish and Wildlife Service Interior Region 9, 3502 Highway 30, La Grande, OR 97850 USA; 5 U.S. Geological Survey, Western Ecological Research Center, Dixon Field Station, 800 Business Park Drive, Suite D, Dixon, CA 95620 USA; 6 U.S. Fish and Wildlife Service, Interior Region 10, 1340 Financial Blvd, Suite 234, Reno, NV 98502 USA; 7 U.S. Fish and Wildlife Service, Interior Region 7, 334 Parsley Blvd, Cheyenne, WY 82007 USA; 8 U.S. Fish and Wildlife Service, Science Applications, 1340 Financial Boulevard, Reno, NV 89502 USA; 9 U.S. Geological Survey, Southwest Climate Adaptation Science Center, USGS at University of Nevada, NV CRU, N Virginia Street, Reno, NV 89557 USA

**Keywords:** Ecosystem ecology, Ecological modelling

## Abstract

We compiled and verified a comprehensive inventory dataset of communication tower infrastructure across the range of the greater sage-grouse (*Centrocercus urophasianus*) and Gunnison sage-grouse (*Centrocercus minimus*), two species of conservation concern that are viewed as ecosystem health indicators for the entire sagebrush biome within the United States. Our dataset includes all known towers with emphasis on validating construction year and month for towers built between 1990–2023. The annual spatial time series format of the data allows users to visualize, assess, and download tower locations and duration (including dates of construction and dismantlement) across the sagebrush biome of the western U.S. Tower data were acquired from publicly available infrastructure databases and records were filtered to include communication tower structures within the area of interest. Data records were validated and checked for accuracy with high resolution aerial and satellite imagery, and a subset were verified during field visits. The final filtered dataset comprises 4,322 tower sites, of which 3,528 tower site locations were verified via satellite imagery or field visits, and 794 were unverified tower sites (tower presence could not be confirmed via satellite imagery). Each tower record includes geographic coordinates, structure height, estimated date of construction, number of towers at each site, and, if applicable, date of dismantlement. The data product closes spatiotemporal gaps and resolves discrepancies present in other public versions of similar data and can be used in ecological research, infrastructure planning/siting/permitting, decision support tools for biological or landscape management, environmental assessments, or general use pertaining to the historic and current locations of communication infrastructure across sagebrush ecosystems.

## Background & Summary

Communication tower infrastructure development increased dramatically in the U.S. during the end of the 20^th^ century and into the 21^st^ century. More than 153,000 cellular towers were operating in the U.S. by 2023. Wireless demand continues to rise, especially in rural areas^1^. The continued development of tower-related infrastructure has posed questions about its influence on wildlife populations, particularly in remote, open landscapes where naturally occurring tall objects are relatively sparse. Towers can have adverse direct effects on avian wildlife^2–4^, and are generally associated with ancillary infrastructure (e.g., roads, powerlines, buildings) that collectively could disturb or degrade habitat for wildlife and plant communities^5–10^. The sagebrush ecosystem within the western U.S. is a vast region characterized by intermixed sagebrush (*Artemisia* spp.), grassland, and other shrubs. Sagebrush plant communities are largely treeless, although in some areas sagebrush may be interspersed with pinyon-juniper woodlands (*Pinus* spp. and *Juniperus* spp) at higher relative elevations. Sagebrush ecosystems are home to many species of conservation concern, including the greater sage-grouse (*Centrocercus urophasianus*) and Gunnison sage-grouse (*Centrocercus minimus*), two species of conservation concern that are viewed as ecosystem health indicators for the sagebrush biome within the United States^11–13^. The sagebrush biome and its wildlife communities may be uniquely affected by the construction of communication tower infrastructure due to being largely void of natural tall features and comprising low human densities and anthropogenic development, relative to other parts of the country. However, while research indicates that tall structures are likely to affect the population dynamics of sage-grouse and other avian species^5,9,14^, high quality spatial data that includes the locations of towers and associated infrastructure has been unavailable. Without these data publicly available at a biome-wide scale, landscape-scale influences on wildlife species cannot be adequately tested.

We initiated research to investigate the potential influences of communication tower infrastructure on sensitive wildlife, such as the sage-grouse, and interactions with avian predator communities. At the onset of the project, we compiled public information on communication tower site locations, and discovered a variety of limitations present in the various datasets that we obtained, such as omission, inaccuracy, missing data (e.g., regarding dates of construction and other attributes), inconsistent reporting, and lack of validation. For these reasons, we developed a methodological workflow to combine all available data sources, verify presence of towers on the landscape, and, where/when possible, provide the estimated date that towers were constructed or dismantled. Our data product represents a comprehensive inventory of communication tower infrastructure between 1990 and July of 2023. It is designed to be a spatiotemporal dataset that allows users to visualize, assess, and download tower locations and duration (i.e., date of construction through date of dismantlement) on western landscapes within the sagebrush biome.

**Fig. 1:**
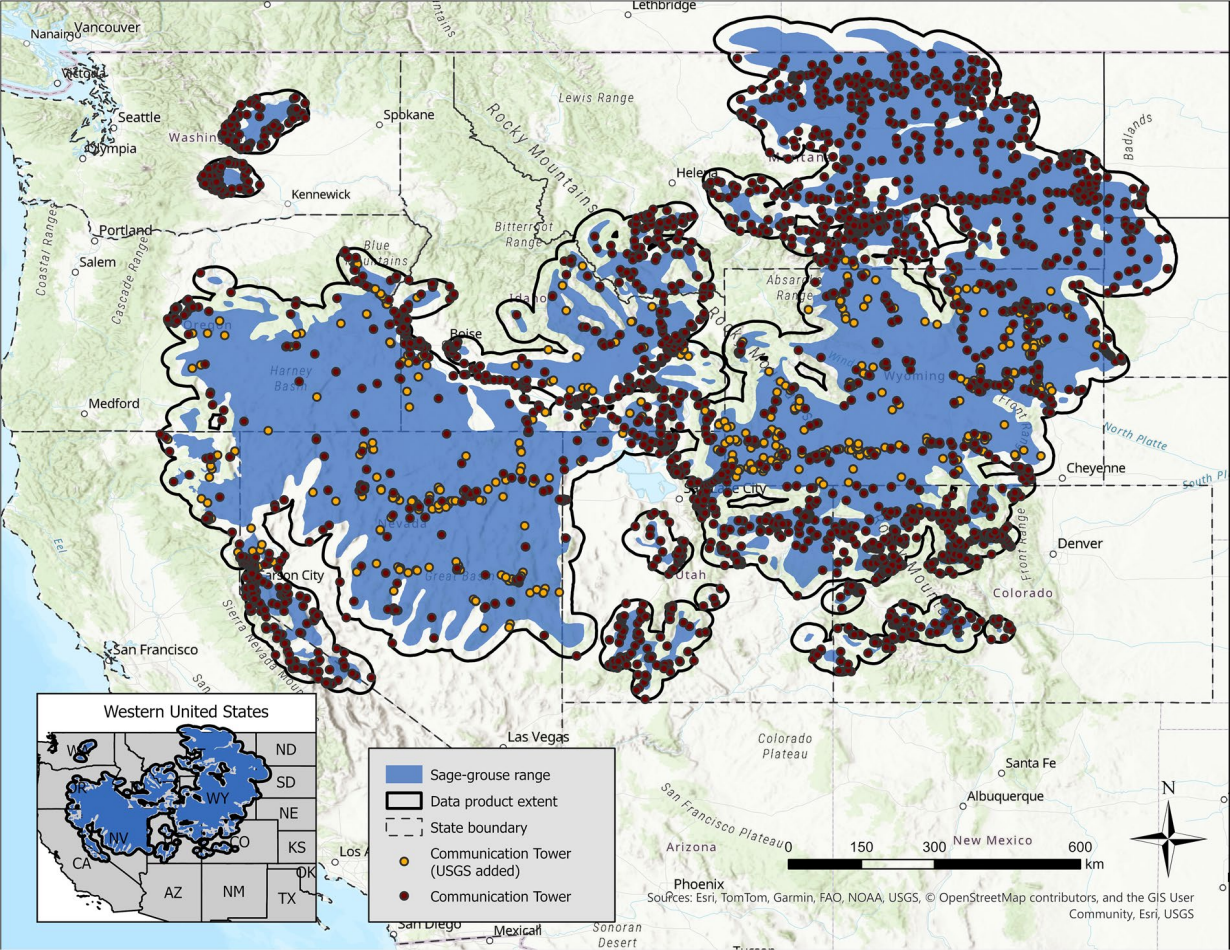
Communication tower infrastructure located within 20 km of the range of the greater sage-grouse (*Centrocercus urophasianus*) and Gunnison sage-grouse (*Centrocercus minimus*)^15,16^ between 1990 and July 2023. Orange points denote towers that were not available in public databases but were found during field surveys.

The data product covers the entirety of the sage-grouse range in the United States^15,16^ with a 20 km buffer, which spans 12 states: California, Colorado, Idaho, Montana, Nevada, North Dakota, Oregon, South Dakota, Utah, Washington, and Wyoming (Fig. 1). Our region of interest comprises sagebrush interspersed with several other vegetation communities including desert shrubland, grasslands, montane forests, and mesic wetlands^17^. Climate and topographical characteristics varied substantially throughout the region, and a broad diversity of land uses were represented.

**Fig. 2:**
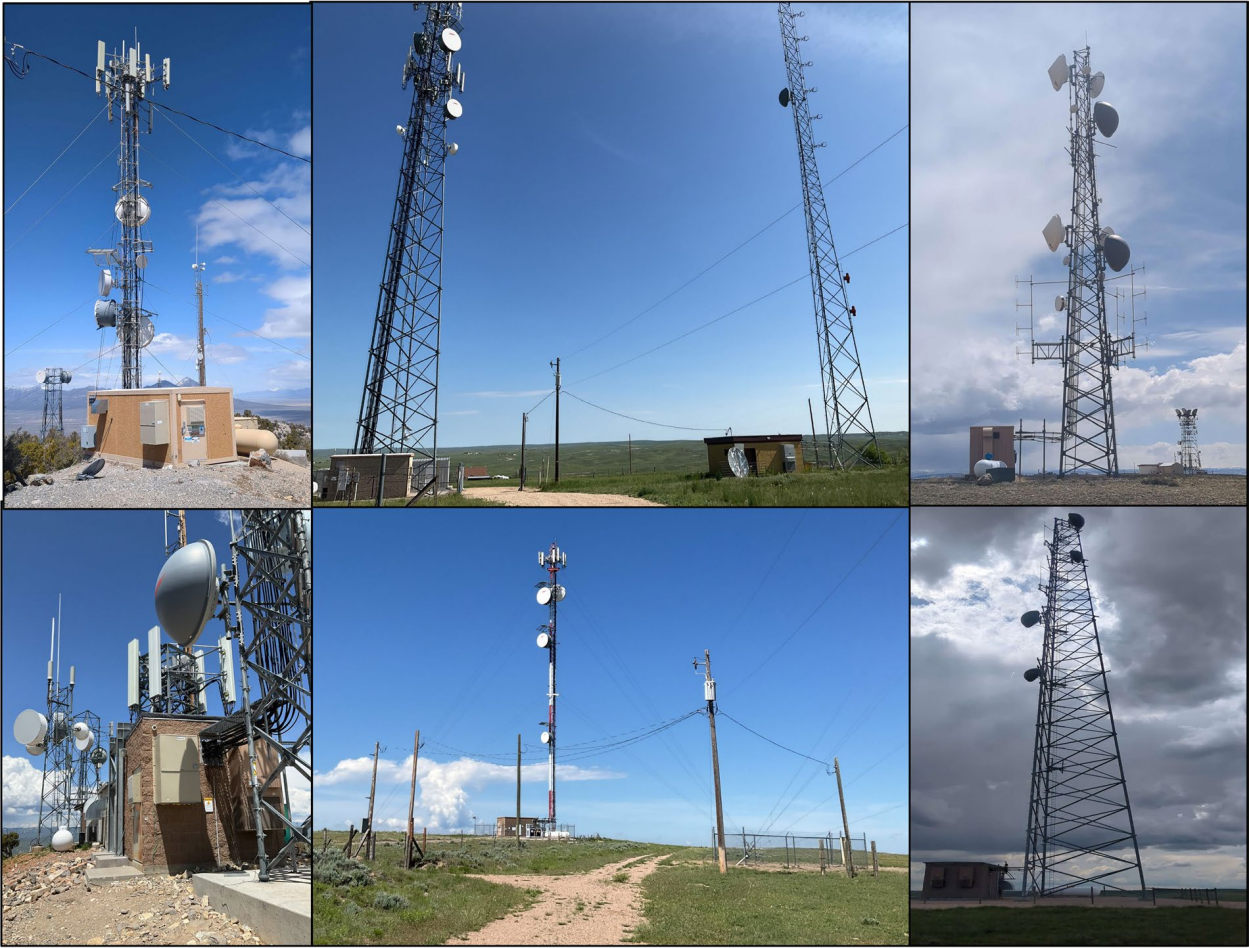
Examples of communication tower sites visited and verified in western sagebrush ecosystems. Characteristics of communication tower sites varied widely across the region, but commonly included one or more towers with varying arrays of antennas, an access road, generator(s) and equipment shelter(s), a fence around equipment, and a power line. All photos are credited to Oregon State University and the U.S. Geological Survey.

We compiled 14,380 total records of communication towers across the product extent of interest. Our final dataset includes 4,322 total records, of which 3,528 were verified tower site locations, occasionally with multiple towers present at the same site. There were 10,058 duplicate records (i.e., multiple records of the same tower site, either for the same tower across data sources or records for different tower structures or antenna infrastructure at a single tower site), that we merged into single verified tower locations in the final data product. A further 794 records were found to not have a tower present at the recorded location during the desktop verification process. We included these records in the final data product as “unverified” tower locations that should be treated with caution if used for analyses, resulting in 4,322 total records (3,528 verified) retained from the original 14,380. We found that tower sites commonly had one tower present (median = 1 tower) at a single location, and we observed sites with up to 14 towers (range = 1–14). Most sites had only one tower present (~85% of verified tower sites, *n = *511), while ~7% of verified sites (*n = *241) had more than two towers, and ~4% of verified sites (*n = *133) had more than three towers. Only 1,078 verified records had date of construction information associated with the original record, and the remaining 2,450 records had date of construction approximated based on when the tower first appeared on available satellite imagery (*n = *2,447) or did not have sufficient information to approximate construction date (*n = *3). Importantly, while there was substantial overlap in tower records among the four databases included in compilation, each database had a substantive number of unique, verified tower records not included in the other databases. Visual examples of communication tower sites are shown in Fig. 2.

Our final data product is formatted as a GIS shapefile. For each tower site in the final data product, we included the following attributes: Record Identification Number, Latitude, Longitude, City, State, Data Source, USGS Verified, FAA Verified, Tower Present, Number of Towers Present, Tower Height (meters), Structure Type, Date of Tower Absence, Date of Tower Appearance/Construction, Date Tower Dismantled, General Notes, and Date Source (Supplementary Table S1). We included some attributes as multiple attribute fields to allow for differences in formatting/units (e.g., geographic coordinates are included twice as both decimal degrees and degrees-minutes-seconds [DMS] to allow the user to select the format most convenient for their purposes).

These data provide improved high accuracy information regarding communication tower presence and site location across the sagebrush ecosystem, closing spatiotemporal gaps and resolving discrepancies present in other public forms of the data. Data can be used to understand and quantify the influence of communication infrastructure on greater sage-grouse and other sensitive fauna within sagebrush ecosystems. Researchers, land managers, landowners, government agencies (federal, state, and local), and the public can all benefit from the dataset that is in downloadable, ready-to-use formats for increased accessibility. Data can also be readily included in ecological research, infrastructure planning/siting/permitting, decision support tools for biological or landscape management, environmental assessments, or general use pertaining to the historic and current locations of communication infrastructure across sagebrush ecosystems.

## Methods

### Tower site locations

**Fig. 3:**
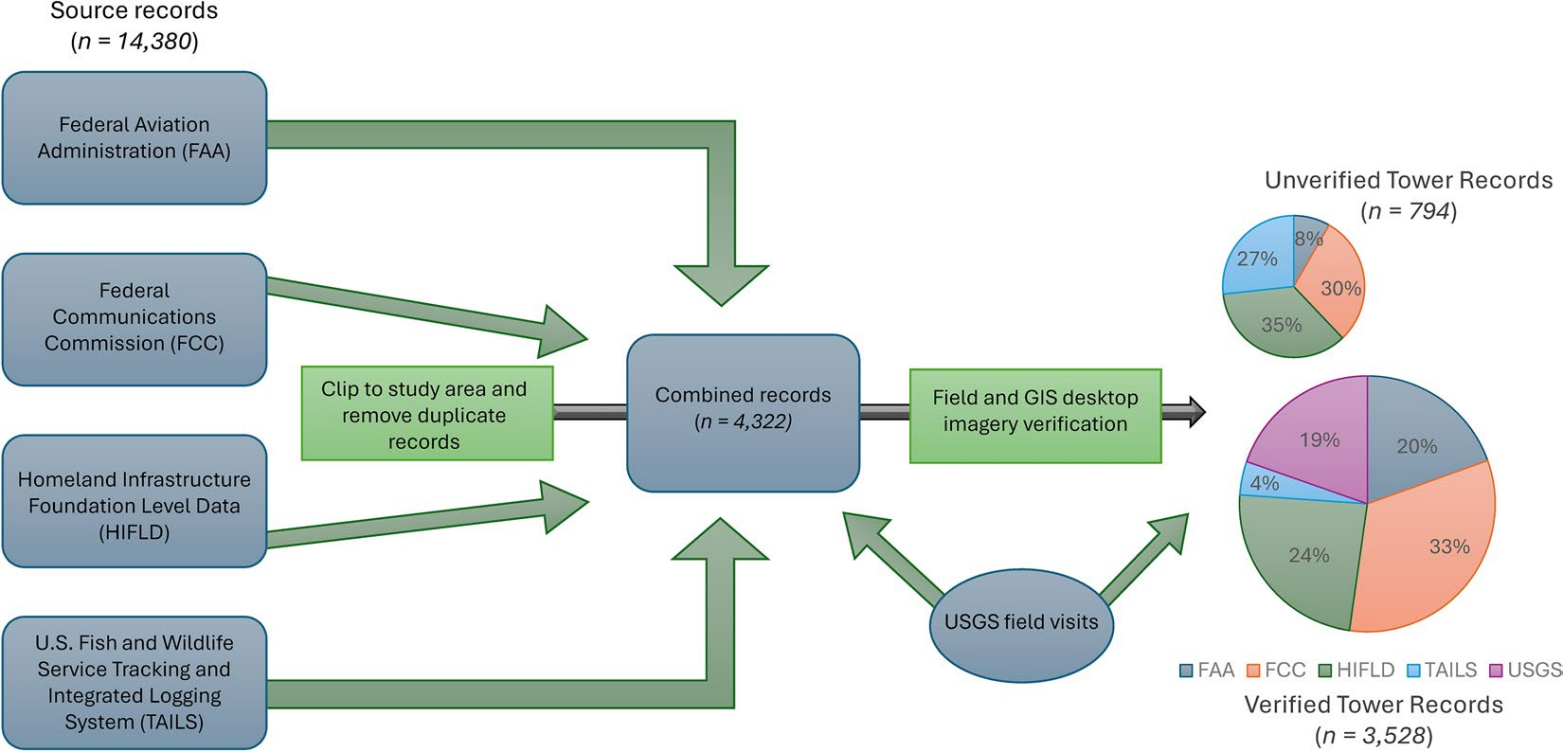
Flowchart diagram demonstrating compilation and verification of communication tower records gathered from multiple sources. Source records were clipped to the study area extent, evaluated for duplicate listings across sources, and verified either by desktop examination of high-resolution satellite imagery (i.e., Google Earth), field site visits, or both.

To create a comprehensive database of communication tower structures across the study area and over time, we obtained potential communication tower locations and year of construction from multiple databases maintained by federal agencies, including the Federal Communications Commission^18^, the U.S. Homeland Infrastructure Foundation Level Data (HIFLD)^19^, the U.S. Federal Aviation Administration (FAA)^20^, and the U.S. Fish and Wildlife Service (FWS; unpublished protected data, Supplementary Table S2, Fig. 3). For each database, we queried all records for states that overlapped the range of sage-grouse^15,16^. For FCC data, we performed a search for all records, state by state. For HIFLD data, we searched for ‘tower,’ and then downloaded each relevant sub-dataset, e.g. ‘cellular tower,’ ‘microwave service tower,’ etc. (see Supplementary Table S2 for comprehensive list). For FAA data, we downloaded the ‘Digital Obstacle File,’ which includes all obstacles of interest to aviation users in the U.S. For FWS data, we searched for all ‘tower’ projects present in the dataset. After the initial downloads were uploaded into a Geographic Information System (GIS; ArcGIS Pro v3.0, Redlands, CA: Environmental Systems Institute [Esri]), we clipped and merged all resulting records, only including records that fell within a 20-km buffer of the delineated sage-grouse range. We chose a 20-km buffer to facilitate use of the database in broad scale analyses, and to accurately capture spatial scales of relevance to the question or species of interest^21,22^. Because our product was targeted towards conservation of the sagebrush biome^13^, we chose a buffer that would capture biologically relevant distances to sage-grouse and avian predators (e.g., common ravens; *Corvus corax*) movement patterns^5,23,24^. We considered a location to have a verified tower if at least one stand-alone, vertical structure (e.g., monopole, lattice tower) was visible within 100 m of the source record location, based on desktop verification. For verified towers without source record information on the year of construction, we approximated the year using time-series imagery from Google Earth (Google Inc., Mountain View, California, U.S.A.) during the data validation process (see *Desktop Verification* section below).

### Desktop verification

For each tower record, we verified the presence of at least one communication tower using available satellite imagery from Google Earth, as evidenced from the appearance of at least one vertical structure, which were often accompanied by their shadow, a road, and additional infrastructure. We then used historic time-series of satellite imagery to determine, to the extent possible, the approximate year the tower was constructed or first appeared on the landscape. For example, if imagery available for a location were dated 2011 and 2014, and a tower site was visible in 2014 (but not 2011), the year of construction was assigned to 2014. We then checked available images for years following, to confirm the tower infrastructure remained present and was not dismantled. Due to limitations in available imagery, if no tower was visible on satellite imagery, we recorded the absence of towers for that site and the record was included in the final data product as an “unverified” tower location. In some cases, a single tower site location had multiple tower records associated with it, either because multiple towers or communication antennae were present at the site, or because the tower site record was found in multiple queried databases, which led to duplicate records when combined. When this occurred, we merged duplicate records and records for multiple towers at a single location into a single record of the site where we recorded the number of towers present, as well as construction year, height, and ownership information for the oldest tower present. Combining the data in this way resulted in a tower record spatial database that demonstrated the approximate year of first alteration at a tower location as well as the magnitude of the alteration (i.e., represented by number of unique towers). The resulting data product thus incorporates a concise spatiotemporal format that is well-suited for inclusion in typical ecological analyses, as opposed to multiple records of a single duplicated tower site which could lead to issues such as false positive errors, double-counting, or pseudoreplication. In a few cases, towers were verified as present during a particular time period on the landscape but were dismantled prior to 2023. When this occurred, we verified the tower record as having a tower present and then also recorded the year the tower was dismantled. This allows users to censor dismantled towers from analyses once they are no longer present on the landscape.

## Data Records

Data represent a comprehensive inventory of communication tower infrastructure across the range of the greater sage-grouse from 1990–2023 (Table 1). The data are in the form of an annual spatial time series product. Tower data were acquired from four publicly available infrastructure databases, filtered within the spatial extent of interest. Data include the locations of 3,528 tower sites, verified via satellite imagery or field visits, as well as an additional 794 records of unverified towers sites (4,322 total records). Data files include attributes that may be useful for filtering and/or sub-setting data for specific cases or analyses, and are available in .csv and .shp (shapefile) formats. Data and associated metadata are publicly available at 10.5066/P14MUQBS^25^.

**Table 1:** Summary of communication tower infrastructure data records.

Subject	Environmental Science/Ecology
**Specific subject area**	Inventory of a common anthropogenic infrastructure constructed between 1990 – July 2023 across a sensitive species’ range.
**Data format**	Filtered and analyzed data in .shp format (Note: raw data were filtered and validated to remove duplicate records and complete data quality and accuracy checks)
**Type of data**	.csv and .shp file (dataset with labels)
**Data collection**	Data were acquired from four publicly available infrastructure databases (See Data Sources) and field surveys, data records were filtered to only include communication tower structures within the spatial extent of interest. Data records were then validated and checked for accuracy using Google Earth 7.3.4.8573 (Google LLC) and any duplicate records were consolidated.
**Data source location**	Both primary and secondary data were collected for the region encompassing the range of the greater (*Centrocercus urophasianus*) and Gunnison sage-grouse (*Centrocercus minimus*) with a 20 km buffer, which spanned 12 U.S. states: CA, CO, ID, MT, ND, NE, NV, OR, SD, UT, WA, and WY. Primary data were collected via field surveys across the range extent from 2021 – 2023. Secondary data were compiled from four infrastructure databases: 1) the Federal Communications Commission Antenna Structure Registrate (FCC ASR), 2) the Homeland Infrastructure Foundation Level Database, 3) The US Fish and Wildlife Service Tracking and Integrated Logging System (TAILS) Database for infrastructure siting consultations, and 4) the Federal Aviation Administration Digital Obstacle File.
**Data accessibility**	Repository name: Sciencebase Data identification number/DOI or persistent identifier: 10.5066/P14MUQBS Direct URL to data: https://www.sciencebase.gov/catalog/item/66b3fe0fd34eebcf8bb33ea7 Instructions for accessing these data: Publicly available for download at the above URL.

## Technical Validation

We visited a subsample of tower records verified during the desktop validation as part of a separate assessment of predator occurrence at communication tower infrastructure. Towers visited in person were located primarily in CA, ID, NV, OR, and WY during the years 2021–23, however towers in CO, MT, and UT were also visited in 2023. During field visits, personnel verified the presence of a tower and attribute information (e.g., height, structure type, presence of ancillary structures, and presence of antennas or other communication infrastructure on the tower structure). We also opportunistically recorded and surveyed towers not captured in desktop data collection/verification. These data were used to conduct accuracy assessments for our tower dataset, allowing us to record false negative (i.e., tower was present but not captured by desktop compilation and verification) and false positive (i.e., record was included in data product, but no tower was present at the tower site) instances across a large portion of our dataset. Any tower records we found during field efforts that were not included in our desktop verification were added to the final tower data product. Tower records that did not have a tower present at field visits were assumed to be dismantled in the final data product, with date of deconstruction approximated based on last evidence of presence in the imagery. Finally, in some cases, if tower presence was not clear from imagery and field visits did not observe a tower, the record was removed from the final product.

During data curation and validation, we found limitations in the available satellite imagery used to verify the presence and duration of towers on the landscape. Overall, desktop data compilation and verification processes only failed to capture 6.1% of communication towers on the landscape (i.e., false negative rate) based on our field verification processes. Conversely, we found 12.02% of communication towers that we visited during field efforts, including both desktop-verified and unverified records, did not exist upon field survey (i.e., false positive rate). Of the tower sites found to not have a tower present (*n* = 124), we determined the majority were “unverified” records (*n* = 79) where towers could not be confidently identified via satellite imagery, while only 45 records were verified as having a tower present in satellite imagery, but no tower was present when surveyed in the field. This discrepancy may be due to imagery limitations (e.g., tower dismantled between last available satellite image and field visit) or misidentification (e.g., pole or other infrastructure mistaken for a communication tower in the satellite image). Based on these findings, we are confident that desktop verification processes we used captured communication tower infrastructure across the entire range of the sage-grouse with relatively high accuracy, which was one of the study’s primary objectives. Further, the identified shortcomings of the desktop verification likely stem from limitations in data availability both in source databases as well as satellite imagery used for verification. The source databases differ in how data is inputted within them. For example, structures that employ communications technologies (e.g., antennae used for radio, broadcast, cellular, and other forms of communication) are legally required to be registered with the FCC prior to construction and updated at the time of construction and time of being dismantled^26^. The FAA, separately, requires that any vertical structure over a height of 45.7 m be registered in their digital obstacle file database^27^, however registration is dependent on self-reporting by landowners or tower owners/operators^27^, particularly for older structures not previously subject to these requirements. As such, the number, type, and characteristics of structures included across different databases would be expected to vary, and we minimized these inconsistencies by compiling data from all available data sources and coalescing any duplicate records, which resulted in a more comprehensive data product than what has previously been publicly available.

Additionally, the temporal range of satellite imagery for desktop verification was not uniform across the data product spatial extent. For example, the first available imagery (oldest available images) that had high enough resolution to discern a tower footprint and structure ranged from 1989 – 2004, while the last available imagery (most recent image) ranged from 2014 – 2023. Thus, it is possible that the desktop verification excluded towers that could not be verified due to imagery gaps/limitations, particularly those tower sites that were constructed after the last available satellite image during verification. Additionally, it is possible that a tower was constructed and then dismantled in the years between available imagery and thus we were unable to verify its presence. For this reason, we included data records that we were unable to verify via satellite imagery within the data product as “unverified” records that may be verified in the future as more imagery data becomes available. Our findings suggest that unverified records in our data product be treated with an abundance of caution and, if possible, verified via a separate method prior to inclusion in analyses.

## Usage Notes

Please refer to USGS Sciencebase 10.5066/P14MUQBS^25^ and associated metadata for appropriate usage.

## Supplementary information

Supplementary Table S1 and S2

## Data Availability

Data and associated metadata are publicly available at the USGS Sciencebase repository, 10.5066/P14MUQBS^25^. Data represent communication tower infrastructure across the range of the greater sage-grouse from 1990–2023 (Table 1). The data can be used to represent an annual spatial time series and include the locations of 3,528 tower sites, verified via satellite imagery or field visits, as well as an additional 794 records of unverified towers sites (4,322 total records). Data files include attributes that may be useful for filtering and/or sub-setting data for specific cases or analyses and are available in .csv and .shp (GIS shapefile) formats.

## Code availability

We did not use custom code in the production of this dataset.
