# Supplementary material for: A comprehensive inventory of communication tower infrastructure across the range of greater and Gunnison sage-grouse
Source: Sci Data. 2026 May 2;13:998. doi: 10.1038/s41597-026-07296-y (PMC13341788; doi:10.1038/s41597-026-07296-y)
Supplement: Supplementary file 1 — Supplementary Table S1 and S2 [file 41597_2026_7296_MOESM1_ESM.pdf]

Supplementary Tables accompanying the article:

Webster, S. C., S. Szabo, J. B. Cupples, S. T. O'Neil, J. B. Dinkins, S. Abele, J. M. Hill, J. C. Tull, M. P. Chenaille, and P. S. Coates. 2026. A comprehensive inventory of communication tower infrastructure across the range of greater and Gunnison sage-grouse. Scientific Data.

Table S1. Communication tower data product attribute fields, descriptions, definitions, and units

ATTRIBUTE NAME	DESCRIPTION	DATA TYPE	ACRONYM DEFINITIONS	UNITS	FORMAT CLASS
**FID_USGS**	Unique Feature Identifier for each tower location	Description	NA	NA	Numeric
**USGSVERIFY**	USGS verified status — Categorical variable denoting whether the tower record has been verified as accurate by USGS officials	Description	Y = Verified; U = Unverified	NA	Text
**STRUCTURES**	Number of tower structures present at tower location	Count	NA	NA	Numeric
**STR_TYPE**	Structure Type — Type of tower present	Description	GTOWER = Guyed structure used for communication; LTOWER = Lattice tower; MTOWER = Monopole tower; NNGTANN = Guyed tower array; NNLTANN = Lattice tower array; NNMTANN = Monopole tower array; NNTANN = Antenna tower array; NTOWER = Multiple tower structures; TOWER = unspecified tower structure; UTOWER = Unguyed free standing structure	NA	Text

**SOURCE**	Original data source of tower record	Description	HIFLD = Homeland Infrastructure Foundation Level Data; FCC = Federal Communication Commission Antenna Structure Registry; FAA = Federal Aviation Administration Digital Obstacle File; TAILS = U.S. Fish and Wildlife Service Tracking and Integrated Logging System	NA	Character
**nLAT**	Geographic coordinate for Latitude	Spatial Coordinates	NA	Decimal Degrees	Numeric
**nLON**	Geographic coordinate for Longitude	Spatial Coordinates	NA	Decimal Degrees	Numeric
**–LAT_DEG**	Latitude Degrees — Partial geographic coordinate for Latitude	Spatial Coordinates	NA	Degrees	Numeric
**–LAT_MIN**	Latitude Minutes — Partial geographic coordinate for Latitude	Spatial Coordinates	NA	Minutes	Numeric
**–LAT_SEC**	Latitude Seconds — Partial geographic coordinate for Latitude	Spatial Coordinates	NA	Seconds	Numeric
**LAT_CD**	Latitude Cardnal Direction — Partial geographic coordinate for Latitude	Spatial Coordinates	N = North	NA	Character
**–LON_DEG**	Longitude Degrees — Partial geographic coordinate for Longitude	Spatial Coordinates	NA	Degrees	Numeric
**–LON_MIN**	Longitude Minutes — Partial geographic coordinate for Longitude	Spatial Coordinates	NA	Minutes	Numeric

**LON_SEC**	Longitude Seconds — Partial geographic coordinate for Longitude	Spatial Coordinates	NA	Seconds	Numeric
**LON_CD**	Latitude Cardnal Direction — Partial geographic coordinate for Longitude	Spatial Coordinates	W = West	NA	Character
**STATE**	Name of U.S. State associated with tower location	Description	NA	NA	Character
**STATE_CODE**	Acronym of U.S. State associated with tower location	Description	NA	NA	Character
**STR_HEIGHT**	Structure Height — Vertical height of communication tower in meters	Description	NA	Meters	Numeric
**HIFLD_PRES**	Yes/No descriptor of whether the tower record was present in the HIFLD database	Description	Y = Yes; N = No	NA	Character
**TAILS_PRES**	Yes/No descriptor of whether the tower record was present in the TAILS database	Description	Y = Yes; N = No	NA	Character
**FCC_PRES**	Yes/No descriptor of whether the tower record was present in the FCC database	Description	Y = Yes; N = No	NA	Character
**FAA_PRES**	Yes/No descriptor of whether the tower record was present in the FAA database	Description	Y = Yes; N = No	NA	Character
**YR_ABS**	Most recent satellite imagery year in which tower is observed absent at tower location prior to its construction	Description	NA	Date	Numeric
**MTH_ABS**	Most recent satellite imagery month in which tower is absent at tower location prior to construction	Description	NA	Date	Numeric

**YR_BUILT**	Year tower was constructed at tower location, or in cases where construction data not available it is the oldest year of satellite imagery in which tower is present at tower location	Description	NA	Date	Numeric
**MTH_BUILT**	Month tower was constructed at tower location, or in cases where construction data not available it is the oldest month of satellite imagery in which tower is present at tower location	Description	NA	Date	Numeric
**YR_DSM**	Appoximate Year Dismantled – – If applicable, year of satellite imagery where tower is no longer visible on imagery post-construction				
**RAILROAD**	Yes/No descriptor of whether tower is associated with railroad track exchanges, crossings, or other railway infrastructure	Description	Y = Yes; N = No	NA	Numeric
**LAST_YR**	Year of most recent satellite image available at the time of validation	Description	NA	Date	Numeric
**LAST_MTH**	Month of most recent satellite image available at the time of validation	Description	NA	Date	Numeric

**NOTES**	General Notes relating to the tower, tower site, surrounding area, etc.	Description	NA	NA	Character
**DATESOURCE**	Indicates the source of information used to approximate date of construction	Description	FAA = FAA; FCC = FCC; GE Imagery = Google Earth Imagery, GSV Imagery = Google Street View Imagery, GE Imagery (No tower) = Google Earth Imagery, no tower verified	NA	Text

Table S2. Databases used to compile communication tower data records across the range of the greater sage-grouse.

SOURCE NAME	DESCRIPTION	SPATIAL EXTENT	DATE ACCESSED	TEMPORAL RANGE (IF APPLICABLE) AT TIME OF ACCESS	URL (IF APPLICABLE)
**FEDERAL COMMUNICATION COMMISSION (FCC) WIRELESS TELECOMMUNICATION BUREAU'S ANTENNA STRUCTURE REGISTRY**	Antenna Structure Registry of registered telecommunications antennas within United States	U.S.	January 12, 2023	Inclusive up to Jan 12, 2023	https://wireless2.fcc.gov/UlsApp/AsrSearch/asrRegistrationSearch.jsp
**FEDERAL AVIATION ADMINISTRATION'S DIGITAL OBSTACLE FILES (DOF)**	Database of vertical structures relevant to air traffic and airspace	U.S.	March 9, 2023	Inclusive up to Nov 27, 2022	https://www.faa.gov/air_traffic/flight_info/aeronav/digital_products/dof/
**HOMELAND INFRASTRUCTURE FOUNDATION LEVEL DATA (HIFLD) BROADBAND TOWERS**	Database of radio broadband towers within the U.S.	U.S.	March 14, 2023	Inclusive up to Sept 9, 2022	https://www.dhs.gov/gmo/hifld
**HIFLD CELLULAR TOWERS**	Database of cellular towers within the U.S.	U.S.	March 14, 2023	Inclusive up to Sept 9, 2022	https://www.dhs.gov/gmo/hifld
**HIFLD FREQUENCY MODULATION (FM) RADIO TOWERS**	Database of FM Radio Towers within the U.S.	U.S.	March 14, 2023	Inclusive up to Sept 9, 2022	https://www.dhs.gov/gmo/hifld
**HIFLD LAND MOBILE FM TOWERS**	Dataset of land mobile FM radio towers within the U.S.	U.S.	March 14, 2023	Inclusive up to Sept 9, 2022	https://www.dhs.gov/gmo/hifld

**HIFLD LAND MOBILE COMMERCIAL BROADCAST TOWERS**	Dataset of commercial broadcast towers within the U.S.	U.S.	March 14, 2023	Inclusive up to Sept 9, 2022	https://www.dhs.gov/gmo/hifld
**HIFLD MICROWAVE SERVICE TOWERS**	Dataset of microwave towers within the U.S.	U.S.	March 14, 2023	Inclusive up to Sept 9, 2022	https://www.dhs.gov/gmo/hifld
**HIFLD TRANSMISSION TOWERS**	Dataset of transmission towers within the U.S.	U.S.	March 14, 2023	Inclusive up to Sept 9, 2022	https://www.dhs.gov/gmo/hifld
**HIFLD TELEVISION ANALOG TOWERS**	Dataset of analog TV towers within the U.S.	U.S.	March 14, 2023	Inclusive up to Sept 9, 2022	https://www.dhs.gov/gmo/hifld
**HIFLD TELEVISION DIGITAL TOWERS**	Dataset of digital TV towers within the U.S.	U.S.	March 14, 2023	Inclusive up to Sept 9, 2022	https://www.dhs.gov/gmo/hifld
**U.S. FISH AND WILDLIFE (USFWS) TAILS DATABASE**	Database of proposed infrastructure seeking USFWS consultation on siting, permitting, or construction	Department of the Interior Regions 1,7, and 9	March 23, 2023	1980 - March 23, 2023	
